# Symbiosis Plasmids Bring Their Own Mutagen to the Wedding Party

**DOI:** 10.1371/journal.pbio.1001943

**Published:** 2014-09-02

**Authors:** Roland G. Roberts

**Affiliations:** Public Library of Science, Cambridge, United Kingdom

[Fig pbio-1001943-g001]Entering into a symbiotic relationship is not something to be taken lightly. Previously free-living organisms become beholden to each other and have to tolerate invasion of their personal space to accommodate varying degrees of intimacy, especially when one partner lives within the body or even the cells of the other. Symbiosis brings with it so many potential mutual benefits that it has arisen independently many times in evolution, but overcoming these barriers requires some encouragement.

**Figure 1 pbio-1001943-g001:**
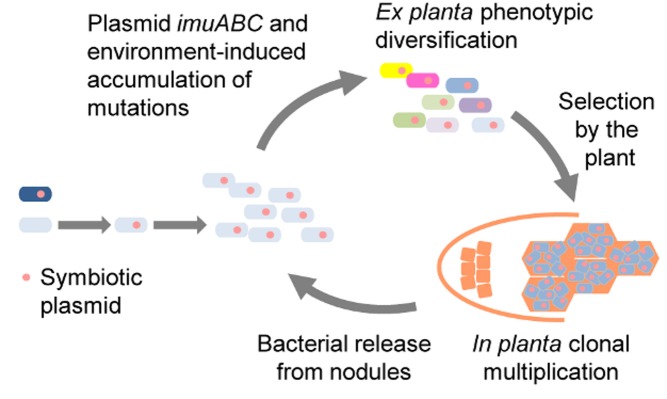
Rhizobial symbiotic plasmids bring the tools to facilitate the symbiotic evolution of the recipient genome. They provoke a burst in genetic diversity, and the plant selects and multiplies the best bacteria.

Among bacteria this independent emergence of symbiosis can be encouraged by the horizontal transfer of “symbiosis plasmids”—DNA-encoded toolkits that help the recipient bacterium acquire the crucial attributes needed to abandon the single life and settle down with a symbiotic host. A classic example of this transition is the establishment of mutualistic relationships between “rhizobial” bacteria and the roots of leguminous plants. Here a taxonomically diverse range of free-living bacteria have acquired the ability to infect plant root cells, to trigger the formation of nodule organs, and to fix nitrogen from the atmosphere that can then be used for biosynthesis by bacteria and plants alike. As a *quid pro quo*, the plant provides other nutrients and shelter to the bacteria.

Although the symbiosis plasmids might be expected to magically confer symbiotic properties on any bacteria that happens to encounter them, in practice, the horizontal transfer of symbiosis is quite hard to achieve in the lab between distantly related species. This perhaps suggests that the recipient bacteria need to undergo further adaptions, beyond the acquisition of a plasmid, in order to go down the symbiotic route.

Catherine Masson-Boivin's group has previously published a study in *PLOS Biology* in which they identified some of the adaptations needed. They took a non-symbiotic soil bacterium *Ralstonia solanacearum* (a pathogen that causes wilt in a wide range of plants) and gave it the symbiosis plasmid from the symbiotic bacterium *Cupriavidus taiwanensis*, which forms nodules on the leguminous plant *Mimosa pudica* (more poetically known as touch-me-not). Despite carrying the genes needed to fix nitrogen and to send seductive signals to the host plant, the plasmid wasn't sufficient to allow the new bacteria to set up a symbiotic relationship with *Mimosa*. Instead, this depended on the rare acquisition of further mutations that disrupted *Ralstonia* genes normally involved in virulence, giving rise to spontaneously arising nodulating strains.

In subsequent work, these authors followed up their findings, setting up an elegant experimental evolution study in which they set out to reproduce the continuing adaptive process and watch it in action. They passed three replicates of each of three mutant nodulating chimeric *Ralstonia* bacteria through 17 cycles of alternating culture in liquid plant medium (*ex planta*) and within plant roots (*in planta*). At the end of this process, which involved about 400 generations of bacterial evolution, they found substantial further improvements in the ability of all nine bacterial lines to nodulate *Mimosa*.

Now, in a new *PLOS Biology* article, Philippe Remigi, Masson-Boivin, and colleagues look at the mutation process that gave rise to this increased performance and find some surprises. They sequenced the genomes of the nine evolved bacteria strains and discovered an unexpectedly high number of mutations, given the modest amount of evolutionary time elapsed. They found that this wasn't because the tough selection regime was driving faster fixation of slowly accumulating mutations, or because the bacteria had become permanent “hypermutators.” Instead, by replaying a single cycle of in *ex planta*–*in planta* evolution, they were able to show that the extraordinary crop of mutations arose through a frenzied but short-lived burst of mutagenesis while the bacteria grew *ex planta*.

The authors speculate that the nutrient-poor medium might be triggering a stress-induced mutagenesis programme in the bacteria, resulting in the observed 5-fold increase in mutation rate; addition of *Mimosa* seedlings boosts this to an impressive 20-fold, perhaps suggesting further unknown stresses from the plants. However, this effect wasn't seen in normal *Ralstonia*, suggesting that the alien symbiosis plasmid from *Cupriavidus* must be responsible for the effect in the chimeric strains.

Looking at the sequence of the plasmid, the authors spotted a cluster of potential suspect genes called the *imuABC* cassette. This cassette is widespread in bacterial genomes and is known to mediate stress-induced mutagenesis. It contains three genes, two of which encode error-prone DNA polymerases, and their presence on the symbiosis plasmid looked like a convincing “smoking gun.” The authors show that the cassette is regulated by LexA, a master SOS response transcription factor. Specific addition or disruption of the *imuABC* cassette in the symbiosis plasmid showed conclusively that it is sufficient to confer a 15-fold increase in mutation rates on *Ralstonia*. Interestingly, *Cupriavidus*, which is already well adapted to nodulation, doesn't hypermutate under stress, so may have found a way to silence its *imuABC* cassette.

This all looks very suspicious, but does the *imuABC* cassette actually drive faster evolution? To test this, the authors set up a competition between chimeric *Ralstonia* that differed only in the presence or absence of the *imuABC* cassette and had been evolved independently for five cycles of *ex planta*–*in planta* evolution. The bacteria whose symbiosis plasmid contained the intact cassette easily out-competed their cassette-less brothers in the race for more effective nodulation.

Finally, to explore whether stress-induced mutagenesis was a peculiarity of the *Cupriavidus* symbiosis plasmid, the authors performed a survey of more than 300 sequenced genomes from α- and β-proteobacteria, representing more than 100 genera. Although the mutagenic *imuBC* cassettes were widespread in bacterial chromosomes, cassettes in plasmids were largely confined to the six rhizobial genera, and nearly half of all symbiosis plasmids contained *imuBC* cassettes. Interestingly, some of these cassettes were found to be vestigial, suggesting that their purpose—presumably adapting their host to symbiosis—was fulfilled long ago and they are now resting on their laurels.

While circumstantial, this genomic survey is consistent with a wider prevalence for the phenomenon described here. In short, it seems that symbiosis plasmids not only encode a genetic toolkit for fundamental symbiotic functions, such as signalling and nitrogen fixation, but also the wherewithal to accelerate the evolution of the host bacterial genome and thereby repurpose it more rapidly for symbiosis. Like bringing a bottle of champagne to a wedding party, the plasmid equips the bacterium with the means to erode social barriers.

**Remigi P, Capela D, Clerissi C, Tasse L, Torchet R, et al. (2014) Transient Hypermutagenesis Accelerates the Evolution of Legume Endosymbionts Following Horizontal Gene Transfer.** doi:10.1371/journal.pbio.1001942

